# Eating Disorders and Disordered Eating Behaviors in Cystic Fibrosis: A Neglected Issue

**DOI:** 10.3390/children9060915

**Published:** 2022-06-18

**Authors:** Anastasia Petropoulou, Georgia Bakounaki, Maria G. Grammatikopoulou, Dimitrios P. Bogdanos, Dimitrios G. Goulis, Tonia Vassilakou

**Affiliations:** 1 Department of Nutritional Sciences & Dietetics, Faculty of Health Sciences, International Hellenic University, Alexander Campus, 57400 Thessaloniki, Greece; anastasiapetropoulou1998@gmail.com (A.P.); gwgw.bkn@gmail.com (G.B.); 2 Department of Rheumatology and Clinical Immunology, University General Hospital of Larissa, Faculty of Medicine, School of Health Sciences, University of Thessaly, 40500 Larissa, Greece; bogdanos@med.uth.gr; 3 Unit of Reproductive Endocrinology, 1st Department of Obstetrics and Gynecology, Papageorgiou General Hospital, Medical School, Aristotle University of Thessaloniki, Agiou Pavlou 76, 56429 Thessaloniki, Greece; dgg@auth.gr; 4 Department of Public Health Policy, School of Public Health, University of West Attica, Athens University Campus, 196 Alexandra’s Avenue, 11521 Athens, Greece

**Keywords:** FEV1, anorexia nervosa, bulimia nervosa, pancreatic enzymes, body satisfaction, self-esteem, thinness, DSM

## Abstract

As with the majority of chronic diseases having specific nutrition recommendations, in cystic fibrosis (CF), the emphasis placed on patients regarding their diet and ideal body weight status often increases the risk of developing disordered eating behaviors and by inference, eating disorders (EDs). Body weight appears to be an important concern for patients with CF, with many patients struggling to lose weight. Between sexes, women appear more preoccupied with dieting compared to men, but exhibit a better body image, mainly due to their preference for a lower weight. Several comorbidities appear to change these dynamics, and visibly apparent factors, including scars, ports, and tubes, and the need for supplementary oxygen supply, may also influence body image perception. Disordered eating is usually initiated during a bout of pulmonary infection, with the patient feeling unwell to eat. Regarding the prevalence of EDs, research appears conflicting on whether it is higher among individuals with a CF diagnosis or not. As for comorbidities, anxiety and depression consist of the most common psychiatric diagnoses in CF, also greatly prevalent in EDs. Despite the plethora of studies, non-specific CF tools, small samples, and lack of data regarding important outcomes, including lung health, indicate the need for more research.

## 1. Introduction

Cystic fibrosis (CF) is a chronic disease affecting several aspects of the nutritional status of patients, including appetite, risk for malnutrition, osteopenia, nutrient deficiencies, and malabsorption [1,2,3]. Attaining an adequate body weight is important for achieving improved respiratory health and low infection risk [1]. However, struggles with weight status are common in CF, with patients of younger ages often failing to maintain and gain body weight [4] and older individuals facing overweight and obesity more frequently [5,6].

As with the majority of chronic diseases having specific nutrition recommendations [1,7], in CF, the emphasis placed on patients regarding their diet and ideal weight status often increases the risk of developing disordered eating behaviors and abnormal eating patterns [8]. Disordered eating is often a precursor to the development of an ED, complicating any underlying pathology while disrupting psychosocial functioning. According to the latest (10th) edition of the International Classification of Diseases and the 5th Edition of the Diagnostic and Statistical Manual of Mental Disorders (DSM-V) [9], typical EDs involve anorexia nervosa (AN), bulimia nervosa (BN), binge eating disorder, pica, and more, whereas other feeding and Eds involve mainly atypical Eds (not fulfilling all diagnostic criteria). The burden of Eds is high, contributing to disability, mortality, and increased medical costs [10]. According to Darukhanavala [11], the nature of CF involves a great degree of preoccupation with body weight, diet, and exercise, which inevitably makes the disease a risk factor for the development of an ED. Moreover, pressure from families and medical personnel appears to aggravate further the existing preoccupation with body image and food [12].

Research suggests that patients with CF often fail to meet daily energy requirements [13,14,15]; however, it is unclear if this is due to the high demands of CF pathology, the result of breathlessness during eating, or the residue of disordered eating. The present review aimed to present all available research on disordered eating and Eds among patients with CF and define factors associated with their prevalence.

## 2. Research on Disordered Eating, EDs, and Other Feeding and EDs among Patients with CF

Table 1 describes all available primary research evaluating disordered eating attitudes and practices and the prevalence of EDs. Most studies have adopted a cross-sectional design [16,17,18,19,20,21], with few case-control studies [22,23,24,25,26,27,28] and case reports [8,29,30,31]. Research originates mainly from the UK [8,16,17,18,20,21,22,23,30], the USA [19,25,26,27,31], Canada [24,29], and Australia [28].

The samples included both pediatric and adult patients with CF. When controls were used to compare the characteristics of patients with CF, these were either healthy participants [22,23,26,28] or had a known ED diagnosis [27]. In one study [25], older individuals with CF were compared to younger ones.

AN and BN were the EDs assessed in the research, with Sher and associates [31] presenting a case report of pica and some researchers focusing on atypical EDs. Few studies evaluated disordered eating behaviors in patients with CF.

### 2.1. Outcomes and Measures

A great variety of outcomes were assessed in the relevant literature. Perception of body image or body mass index (BMI) was evaluated using ‘The Body Test’ [59], devised by the Canadian Dietetic Association, the Body Shape Questionnaire [34], the 24-item Body Esteem Scale [62], or the Children’s Body Image Scale [36], and body satisfaction was evaluated with a revised version of the Body Image Questionnaire [33].

Eating attitudes and behaviors were evaluated by employing a variety of tools, including a revised measure of eating pathology, namely the Eating Attitudes Test [45], the Eating Disorders Inventory [47], the Eating Disorder Questionnaire [48], the Eating Disorders Examination [46], the Slade Anorexic Behavior Scale [53], the Child Version of the Eating Disorder Examination [38], and the Children’s Eating Attitude Test [40]. Furthermore, restraint eating was assessed using the Dutch Eating Behavior—Restraint Scale [41] and the Three-Factor Eating Questionnaire—Short Form [60], which evaluates the concepts of restrained eating, emotional eating, and uncontrolled eating. The motives for eating tasty, but unhealthy foods for reasons other than hunger were examined using the Palatable Eating Motives Scale [52].

Self-perception was also evaluated using the self-comparison scale [54] and self-esteem using the Rosenberg Self-esteem Scale [63], or, in pediatric samples, a validated five-item children’s version of the Rosenberg Self-esteem Scale [62] was employed. The Defense Style Questionnaire [44] was applied to evaluate the defense styles of participants, and mindfulness was assessed using the Mindfulness Eating Scale [51] and the Five-Facet Mindfulness Questionnaire—Short Form [49].

The assessment of mental health status was also performed in some studies, using the National Institute of Mental Health Diagnostic Interview Schedule [42] for adults and the Diagnostic Interview Schedule for Children [43]. Furthermore, a behavioral disturbance was assessed in children using the Total Behavior Problem score of the Child Behavior Checklist [35] and in adults via the total score on the 90-item Symptoms Checklist [55]. Anxiety was evaluated in children using the Trait Scale from the State-Trait Anxiety Inventory for Children [58] and using the Trait Scale from the State-Trait Anxiety Inventory [57] or a modified version of the Sheehan Patient-rated Anxiety Scale [56] in adults. For depression, the Child Depression Inventory [37], the Zung Self-Report Depression Inventory [61], the Beck Depression Inventory [32], or the Hamilton Depression Rating Scale [50] were employed.

Physical status was evaluated using the Schwachman–Kulczycki (1958) ratings of activity level, and quality of life (QoL) was evaluated using the CFQoL questionnaire [39].

The variety of tools employed makes it difficult to conclude certain aspects of the symptomatology and pathology associated with DEB and EDs in CF.

### 2.2. Body Weight, Body Image, and BMI

According to Barrett [16], body weight and BMI are important concerns for patients with CF, with many patients struggling to lose weight [17]. Moreover, attaining a body mass within the recommended cut-offs does not appear to predict a healthy diet behavior among youngsters with CF [17]. Children and adolescents with CF report lower body size and weight satisfaction scores, perceiving their body as a little larger than it currently is [28].

Between sexes, women appear more preoccupied with dieting compared to men; however, when compared to a group of healthy women, no differences were noted in the dieting behavior [22,23]. According to Tierney [64], women with CF exhibit a better body image compared to men, mainly due to their preference for a lower body weight and a thinner frame. Men with CF are willing to gain body mass, in contrast to apparently healthy men lacking a CF diagnosis [22,23]. Tierney [64] suggested that given that a larger form is more desired in men in general, male patients with CF may be more motivated to adhere to nutritional advice. 

Nevertheless, several additional comorbidities appear to change these dynamics, and visibly apparent factors, including scars, ports, and tubes, and the need for supplementary oxygen supply, may also influence body image perception [11]. For instance, in CF-related diabetes, women have been dissatisfied with their body appearance, exhibiting low self-esteem [30]. When patients with CF receiving no intervention were compared to those on a nasogastric tube or receiving oral nutrient supplementation, the first exhibited more aggravated dieting behavior [22,23]. According to Meloff [24], pancreatic sufficient patients tend to exhibit greater pathology surrounding body shape and BW concerns compared to the pancreatic insufficient ones.

### 2.3. Disordered Eating Attitudes and Practices in CF

It has been hypothesized that the emphasis placed on patients with CF regarding their diet and body weight could place them at increased risk of developing DE [8]. Disordered eating practices are usually initiated during a bout of pulmonary infection, with the patients feeling “too ill to eat” and hunger denial persisting post-recovery from the infection [19]. 

However, compared to healthy controls, both minors and adults with a CF diagnosis have been shown to exhibit lower disordered eating behaviors. Although children and adolescents with CF have been reported to exhibit less eating restraint compared to their healthy peers [28], often dietary control struggles escalate, fueled mainly by the parental reaction to their refusal to eat [19]. This pressure to eat is a universal finding in CF, irrespective of the age of the patients [22,23]. Moreover, the drive for thinness, perfectionism, and body dissatisfaction subscales are greater among healthy controls as compared to participants with CF [26], indicating that in CF, the importance of attaining a healthy body weight is greatly acknowledged. According to Pearson [25], younger patients are more likely to manifest disordered eating and EDs compared to older patients, as older ones are more experienced in managing CF and have more knowledge regarding the disease.

### 2.4. Prevalence of EDs in Populations with CF

The literature appears conflicting on whether the prevalence of EDs is higher among individuals with a CF diagnosis. Some studies report a greater prevalence of EDs in CF [19,21,25], whereas others report a similar prevalence to that of the rest of the population [26,27,28]. However, due to the small samples used, it is difficult to conclude whether CF diagnosis increases the prevalence of EDs or if the frequent medical visits allow for prompt identification and guidance of patients in need. According to Pumariega [19], when AN and CF coexist, female patients develop amenorrhea and obliteration of secondary sexual characteristics, further delaying puberty. Moreover, the growth of lanugo hair [19] indicates an additional increase in the energy demands to maintain core temperature, making it more difficult to attain a healthy body weight. On the other hand, as Linkson [30] noted, the long time required to recover from AN may exacerbate all CF-related pathologies.

According to Darukhanavala [11], one often overlooked symptom of disordered eating behavior involves the manipulation of body weight through misuse/abuse of glucocorticoids, pancreatic enzyme replacement therapies, or insulin (in CF-related diabetes). Pumariega [19] first identified such practices among patients with CF, aiming to lose or maintain body weight.

### 2.5. Effect of Disordered Eating and EDs on Respiratory Function

Only a handful of studies evaluated forced expiratory volume at 1 s (FEV_1_) among patients with CF demonstrating disordered eating attitudes. Case reports suggest a reduced FEV_1_ among females with CF and AN [8,30]; however, none of the studies using a set of participants with a CF diagnosis have evaluated the effect of disordered eating on respiratory function or risk for respiratory infections. However, a great body of evidence has related low BMI and poor nutritional status in CF to poorer respiratory function [65,66]. Thus, it has been suggested that a two-way relationship appears to exist between nutritional status and pulmonary function, with each one affecting the other and the “obesity paradox” prevailing, as patients with greater BMI have improved prognosis [1].

Regarding respiratory infections, no study has evaluated their frequency concerning disordered eating patterns or ED diagnosis.

In the only case report of pica in CF [31], however, the patient exhibited excellent lung function, having received a transplant a few months prior to the pica and iron deficiency anemia diagnosis.

### 2.6. Comorbidities in CF and Disordered Eating

A psychiatric background is often apparent in CF, with anxiety, major depression, disruptive behavior, and alcohol and/or nicotine dependence prevalent in children and their parents [26,27,67]. Compared to populations without a diagnosis of CF, children with CF and their parents appear to exhibit greater anxiety levels [67]. The global pooled prevalence of anxiety and depression reaches 24.91% [68], with symptoms being more frequent in older patients compared to younger ones [25]. According to Pumariega [19], patients with CF and atypical EDs often demonstrate depression and dysthymia. Given that anxiety and depression are the most common comorbid diagnoses in EDs [69], prompt identification and treatment of these conditions might prove useful in reversing the development of EDs.

## 3. Limitations of Research on Disordered Eating Behaviors and EDs in CF

Despite the large body of evidence evaluating disordered eating behaviors and EDs in patients with CF, many studies are case reports, and the majority of the remaining are biased by using relatively small samples. Moreover, the great variety of psychometric batteries and tools employed, each assessing slightly different outcomes from the others, makes it difficult to conclude the drivers and pathologies associated with disordered eating behaviors and EDs in CF. In parallel, the lack of CF-specific tools for the assessment of disordered eating patterns, except for the CFEAB [20], indicate that several aspects and practices associated with disordered eating in CF may be missed, including the misuse of pancreatic replacement enzyme therapies or insulin. Furthermore, important disease outcomes, including FEV_1_, have not been evaluated in populations exhibiting disordered eating behaviors, and gene mutations associated with more frequent disordered eating practices have not been identified. Last but not least, most of the research originates from developed, English-speaking countries, suggesting that differences might be apparent if research is conducted in other populations.

## 4. Conclusions

Disordered eating behaviors and EDs appear common in CF, although it is unclear if the prevalence is greater or similar to that of the general population. Collectively, the studies suggest that specific factors associated with CF treatment and comorbidities may negatively affect body image perception and the psychological health of patients. Due to the nature of CF, the impact of body weight loss appears to be far more detrimental than other conditions, and thus, patients with unexplained loss of body weight should be identified early on and screened [30]. 

Nevertheless, more research is required, using CF-specific tools and larger samples to assess this important issue. Prolonging health and survival is the cornerstone of medicine and the most important outcome in CF management; thus, routine screening for disordered eating behaviors, other specified feeding, and EDs should be performed promptly to identify patients at risk.

## Figures and Tables

**Table 1:** Primary studies assessing the prevalence of disordered eating, EDs, or other specified feeding and EDs among patients with CF.

First Author	Study Design	Origin	Recruitment		Participants				Outcomes	Diagnostic Criteria and/or Tools	Results Summary
			Year	Site(s)	N	Age (Years)	Females (%)	RR (%)			
Abbott [22,23]	CC	UK	NR	Manchester and Leeds CF units	N = 221 patients with CF and 148 healthy controls	16–51	52.3 CF, 20.05 among controls	76% for CF	Dieting behavior, body satisfaction and perception, QoL, self-esteem, food preoccupation, and pressure from others to eat	EAT, BIQ, TBT, CFQoL	Males with CF wished to be heavier. Females with CF saw themselves as thinner than they were but felt content with their body image. They engaged in less dieting and preoccupation with food than their healthy peers, but demonstrated greater dieting behavior and lower self-esteem than males with CF. A minority of patients reported disordered eating. Those receiving nutritional interventions engaged in less dieting, but all patients were more pressured to eat.
Barrett [16]	CS, qualitative	UK	2017–8	Single specialist UK adult CF center	N = 9 patients with CF	23–48	30	NR	Relationship with food and eating and perception	Semi-structured telephone interviews	Body weight gain, body image, and dietary health implications are important concerns for these patients.
Bryon [17]	CS	UK		Two UK CF centers in London	N = 55 children and adolescents with CF	11–17	49.1	NR	Disordered eating, AN, BN	CEDE	No participant met the criteria for a diagnosis of AN/BN. Few patients met some criteria for an ED diagnosis, though not sufficient to rate a full diagnosis. Most participants had body weight within the desirable BMI range, although some were engaging in behaviors to lose weight (18.8% female, 7.1% male) or avoid weight gain. One male was diagnosed with an USFED, 3% demonstrated disordered eating attitudes, and 16% demonstrated disordered eating behaviors. No sex differences existed.
Egan [18]	CS	UK	NR	Regional Adult CF Centre	N = 92 adult patients with CF	30.8 ± 10.65 *f*	48.9	NR	Mindfulness eating, motivation to eat palatable foods	MES, PEMS, FFMQ-SF	Motivations to eat palatable foods and eating behaviors correlated with greater BMI. Mindfulness and mindful eating moderated the relationship between emotional eating and BMI.
Gilchrist [8]	CR	UK	2008	North Staffordshire University Hospital	N = 1	15	100	N/A	Distorted body image and AN	NR	The patient highlights the importance of nutritional status in CF, revealing how complex it often is for the CF team to assist.
Goldbloom [29]	CR	Canada	1985	Toronto General Hospital	N =1 patient with CF and AN	24	100	N/A	AN, EDI	NR	The medical complications of the ED included chronic hypokalemia, episodic weakness, bilateral parotid hypertrophy, post-prandial abdominal bloating, and pain. The patient had multiple medical admissions and invasive investigations for abdominal pain that followed bulimic episodes. Post-binges she often vomited blood.
Linkson [30]	CR	UK	2018	Adult CF Service	N = 1 female patient with CF and AN	20	100	N/A	AN	NR	BW loss is far more significant than in a healthy population.
Meloff [24]	CC	Canada		Foothills Medical Centre Adult CF Clinic, Calgary	N = 34 patients with CF and 44 healthy controls	18–41	NR	NR	Disordered eating behaviors	EAT, EDI, EDE	Patients with CF exhibited ED symptomatology. Those who were pancreatic sufficient had greater pathology surrounding body shape and body weight concerns than their insufficient pancreatic counterparts.
Pearson [25]	CC	USA	1991	Pediatric Pulmonology section, Baylor College of Medicine and Junior League Clinic, Texas Children’s Hospital	N = 61 children with CF (group 1) and 36 older individuals with CF (group 2)	Group 1: 8–15 Group 2: 16–40	Group 1: 44.2 Group 2: 44.4	NR	EDs, anxiety, depression and general behavioral and emotional disturbance	EAT, STAIC, STAI, CDI, ZSRDI, CBCL, SCL-90	Approximately 16.4% of the younger patients reported symptoms consistent with AN, compared to 2.8% of the older group. Younger patients were more likely to manifest eating disturbances (resisting food, being preoccupied with food, using food for control) than older patients.
Pumariega [19]	CS	USA	NR	NR	N = 13 patients with CF and EDs	12–21	76.9	N/A	Atypical EDs, depression, dysthymic disorder	DSM-III	Seven patients met the diagnostic criteria for a depressive disorder, while six met the criteria for dysthymic disorder. Several patients developed amenorrhea and obliteration of secondary sexual characteristics (girls), muscle wasting, and lanugo, exhibiting an increasing preoccupation with food and hunger denial.
Randlesome [20]	CS	UK	NR	3 hospital CF clinics (2 pediatric, 1 adult) at 2 Specialist CF Centers, London	N = 155 patients with CF	11–62	52.3	NR	EABs	CFEAB	PCA revealed a 3-factor structure, with one regarding the ‘Desire for thinness and BW loss’, one for ‘Disturbed EABs’, and one for ‘Appetite’.
Raymond [26]	CC	USA	NR	University of Minnesota Hospital CF Center	N = 58 patients with CF and 43 controls	13–20	42	74	Mental health status, depression, disordered eating	DSM-III-R, DIS, BSQ, SPAS, BDI, EDI, EDQ, DIS, DISC, HDRS	None of the CF participants met the diagnostic criteria for EDs. The EDI revealed greater scores for controls regarding the drive for thinness, perfectionism, and body dissatisfaction compared to the patients with CF.
Shearer [21]	CS	UK	NR	2 pediatric CF centers	N = 55 adolescent patients with CF	11–17	49.1	NR	AN, BN, OSFED	DSM-IV, CEDE	None of the participants met all the criteria for AN/BN. One male patient met the criteria for OSFED. Some participants met several of the diagnostic criteria for AN/BN.
Sher [31]	CR	USA	NR	NR	N = 1 vegetarian woman with CF	53	100	N/A	Pica, IDA	NR	The patient presented IDA and pica, particularly beeturia (uncooked tofu) and pagophagia.
Steiner [27]	CC	USA	NR	CF clinic, Children’s Hospital at Stanford	N = 10 patients with CF and 10 matching controls with AN *	10–20	100	N/A	EDs, disordered eating, depression, anxiety	DSM-III-R, EDI, SABS, DSQ, BDI, STAI, F-COPES	AN showed more psychopathology, and families of patients with AN employed fewer adaptive coping strategies. Core features of AN (drive for thinness, body dissatisfaction, lack of interoceptive awareness, and disordered eating) distinguished these groups, equally affected by malnutrition and pubertal delay.
Truby [28]	CC	Australia	2001	Royal Children’s Hospital	N = 76 patients with CF and 153 healthy controls	7–12	51.3 CF and 53.6 controls	N/A	Body image and eating attitudes	CBIS, Rosenberg Self-Esteem Scale, 24-item Body Esteem Scale, DEBQ–R, ChEAT	No differences were noted between groups or sex regarding body esteem. Children with CF were more likely to perceive their ideal body size as slightly larger than their current one and had a lower score for body size and weight satisfaction. The CF arm had lower scores on the DEBQ–R scale.

AN, anorexia nervosa; BDI, Beck Depression Inventory [32]; BIQ, Body Image Questionnaire [33]; BMI, body mass index; BN, bulimia nervosa; BSQ, Body Shape Questionnaire [34]; CBCL, Child Behavior Checklist [35]; CBIS, Children’s Body Image Scale [36]; CC, case-control; CDI, Child Depression Inventory [37]; CEDE, Child Version of the Eating Disorder Examination [38]; CF, cystic fibrosis; CFEAB, cystic fibrosis eating attitudes or behaviors [20]; CFQoL, cystic fibrosis quality of life [39]; ChEAT, Children’s Eating Attitude Test [40]; CR, case report; CS, cross-sectional; DEBQ–R, Dutch Eating Behavior—Restraint Scale [41]; DIS, Diagnostic Interview Schedule [42]; DISC, Diagnostic Interview Schedule for Children [43]; DSM, diagnostic and statistical manual for mental disorders; DSQ, Defense Style Questionnaire [44]; EABs, eating attitudes or behaviors; EAT, Eating Attitudes Test [45]; ED, eating disorder; EDE, Eating Disorders Examination [46]; EDI, Eating Disorders Inventory [47]; EDQ, Eating Disorder Questionnaire [48]; FFMQ-SF, Five-Facet Mindfulness Questionnaire—Short Form [49]; HDRS, Hamilton Depression Rating Scale [50]; IDA, iron deficiency anemia; MES, Mindfulness Eating Scale [51]; N/A, not applicable; NR, not reported; OSFED, other specified feeding and eating disorders; PCA, principal components analysis; PEMS, Palatable Eating Motives Scale [52]; QoL, quality of life; RR, response rate; SABS, Slade Anorexic Behavior Scale [53]; SCS, self-compassion scale [54]; SCL-90, 90-item Symptoms Checklist [55]; SPAS, Sheehan Patient-rated Anxiety Scale [56]; STAI, State-Trait Anxiety Inventory [57]; STAIC, State-Trait Anxiety Inventory for Children [58]; TBT, The Body Test [59]; TFEQ-R18, Three-Factor Eating Questionnaire—Short Form [60]; ZSRDI, Zung Self-Report Depression Inventory [61]; * Matched for age, body weight, BMI, BF and puberty; *f* mean ± standard deviation.

## Data Availability

Not applicable.
